# Genotype imputation for the prediction of genomic breeding values in non-genotyped and low-density genotyped individuals

**DOI:** 10.1186/1753-6561-5-S3-S6

**Published:** 2011-05-27

**Authors:** Matthew A Cleveland, John M Hickey, Brian P Kinghorn

**Affiliations:** 1 Genus plc., 100 Bluegrass Commons Blvd., Suite 2200, Hendersonville, TN, 37075, USA; 2 School of Environmental and Rural Science, University of New England, Armidale, NSW, 2351, Australia

## Abstract

**Background:**

There is wide interest in calculating genomic breeding values (GEBVs) in livestock using dense, genome-wide SNP data. The general framework for genomic selection assumes all individuals are genotyped at high-density, which may not be true in practice. Methods to add additional genotypes for individuals not genotyped at high density have the potential to increase GEBV accuracy with little or no additional cost. In this study a long haplotype library was created using a long range phasing algorithm and used in combination with segregation analysis to impute dense genotypes for non-genotyped dams in the training dataset (S1) and for non-genotyped or low-density genotyped individuals in the prediction dataset (S2), using the 14^th^ QTL-MAS Workshop dataset. Alternative low-density scenarios were evaluated for accuracy of imputed genotypes and prediction of GEBVs.

**Results:**

In S1, females in the training population were not genotyped and prediction individuals were either not genotyped or genotyped at low-density (evenly spaced at 2, 5 or 10 Mb). The proportion of correctly imputed genotypes for training females did not change when genotypes were added for individuals in the prediction set whereas the number of correctly imputed genotypes in the prediction set increased slightly (S1). The S2 scenario assumed the complete training set was genotyped for all SNPs and the prediction set was not genotyped or genotyped at low-density. The number of correctly imputed genotypes increased with genotyping density in the prediction set. Accuracy of genomic breeding values for the prediction set in each scenario were the correlation of GEBVs with true breeding values and were used to evaluate the potential loss in accuracy with reduced genotyping. For both S1 and S2 the GEBV accuracies were similar when the prediction set was not genotyped and increased with the addition of low-density genotypes, with the increase larger for S2 than S1.

**Conclusions:**

Genotype imputation using a long haplotype library and segregation analysis is promising for application in sparsely-genotyped pedigrees. The results of this study suggest that dense genotypes can be imputed for selection candidates with some loss in genomic breeding value accuracy, but with levels of accuracy higher than traditional BLUP estimated breeding values. Accurate genotype imputation would allow for a single low-density SNP panel to be used across traits.

## Background

Numerous approaches have been suggested for predicting genomic breeding values (GEBVs) using single nucleotide polymorphisms (SNPs), generally based on the framework described by Meuwissen *et al.*[1]. These approaches often assume that high-density genotypes are available for all individuals, both for estimating marker effects and for predicting the subsequent GEBVs. This assumption may not be valid in some livestock species, such as pigs, where the cost of genotyping selection candidates for many SNPs is cost prohibitive, but low-density panels could be used as they are often much less expensive (A. Mileham, *pers. comm.*). These panels are often comprised of SNPs associated with few traits in a single population, whereas, ideally, they would be applicable across multiple traits and populations. Habier *et al.*[2] suggested an approach for imputing high-density genotypes for low-density genotyped individuals, assuming pedigree information is available and ancestors are genotyped at high density. This type of approach would obviate the need for multiple SNP panels for genomic selection, but assumes a level of high-density genotyping that may be difficult to achieve in practice. An alternative, termed segregation analysis and long haplotype library imputation (SALHI, [3]), involves building a library of long haplotypes for a population by phasing densely genotyped individuals and then inferring haplotypes through the remainder of the population using individual SNP genotype probabilities (e.g., [4]), optionally aided by low density genotypes for some individuals. Meuwissen and Goddard [5] have described a similar approach to impute genotypes in whole genome sequence density data that uses segregation analysis but not the concept of a haplotype library.

This study evaluated an implementation of SALHI in terms of the accuracy of genotype imputation and the accuracy of GEBVs calculated from imputed and non-imputed genotypes, using data simulated for the 14^th^ QTL-MAS Workshop.

## Methods

### Description of imputation

SALHI uses segregation analysis [4] to determine genotype probabilities for ungenotyped individuals in a pedigree and a long range phasing and long haplotype imputation algorithm [6] to phase densely genotyped individuals and build a haplotype library. When low-density genotypes are available these can be used in place of genotype probabilities (geneprobs). Hickey *et al*. [3] match all possible haplotype pairs to the genotype probabilities (and low-density genotypes where known) of each individual and choose the best matching pair of haplotypes using a product of probabilities across loci. The current implementation differs in that a pre-selection step is included where the most probable genotype for each individual is inferred from genotype probabilities (and low-density genotypes) with exclusion of loci without enough information to distinguish between genotypes. Single haplotypes and then haplotype pairs are selected by matching the most probable genotypes, followed by an approach similar to that implemented by Hickey *et al.*[3] if any additional ambiguity in the correct selection remains. Each geneprob has an index (GPI) that indicates its quality of information content [7]. The imputation approach described here combines this information to identify putative haplotypes for each ungenotyped or low-density genotyped individual using the following algorithm:

#### Step 1

The most probable genotype is determined at each locus, based on the geneprob, where the default genotype is the heterozygote when a single geneprob is not more probable than the others (i.e., all genotypes are equally likely). A minimum GPI threshold is selected so that loci with low information content are not used in this step. This threshold is generally set high, but may change based on previous performance in the target population. The most probable genotype at each putative homozygous locus exceeding the GPI minimum is compared to the corresponding locus in each *single* haplotype contained in the library. If the number of opposing loci (i.e., the haplotype allele can not produce the genotype) exceeds a pre-determined error threshold the haplotype is excluded from further consideration.

#### Step 2

A new GPI minimum is selected (which is generally very high) to determine which loci are used in this step. The putative haplotypes that remain from Step 1 are paired in all possible combinations to yield genotypes. Each locus (homozygous or heterozygous) exceeding the GPI minimum is compared to the corresponding locus in each haplotype *pair*. If the number of conflicting genotypes exceeds a pre-determined error threshold the haplotype pair is excluded from further consideration. If more than one putative haplotype pair remains this step is repeated with a lower GPI minimum until one haplotype remains or until a pre-defined lower bound on the GPI minimum is reached.

#### Step 3

If more than one haplotype pair remains after Steps 1 and 2, the probability of each haplotype pair is calculated using the geneprob at each locus as the sum of the products of the geneprob and putative genotype across all loci. The pair with the highest probability is retained.

In each of the first two steps no putative haplotypes or haplotype pairs may be identified but additional functions may identify a proportion of individual genotypes based on the frequency of a genotype in multiple haplotype pairs or when the GPI indicates that there is no ambiguity in the proposed genotype. Additionally, the parameters for the GPI minimums or error thresholds are user inputs and can be adjusted to fit the data to increase the likelihood of identifying putative haplotype pairs.

### Data

The dataset used to evaluate the imputation was simulated as part of the 14^th^ QTL-MAS Workshop, see [8] for details. The data consisted of 3226 individuals in five generations, where each female parent had about 30 offspring. The first four generations had phenotypes for a quantitative trait (N=2326), comprising the training dataset, while the last generation (N=900) had no phenotypes and comprised the prediction dataset for calculation of GEBVs. All individuals were genotyped for 10031 SNP markers, across five chromosomes of length ~100 Mb each.

### Training and prediction sets

The data were partitioned or masked to create three main scenarios to evaluate alternative genotyping strategies. The first scenario (BASE) assumed the general framework for calculating GEBVs, where all individuals were genotyped for all SNPs. In the second scenario (S1) only the males in the training population were genotyped for all SNPs, whereas the females were not genotyped. The individuals in the prediction set were either not genotyped or genotyped for SNPs spaced 2, 5 or 10 Mb apart. The third scenario (S2) had the same training population as BASE and the same prediction population as S1.

### Imputation, training and GEBV prediction

The prediction of GEBVs for S1 and S2, using imputed genotypes proceeded as follows:

1. A haplotype library was created using only males (S1) or all individuals (S2) in the training set. The phasing was performed for 12 sections (or cores) of each chromosome of approximately 10 Mb and then combined to form one long haplotype for each chromosome, using the software package AlphaPhase, being developed based on Hickey *et al.*[6].

2. Genotype probabilities were calculated for training females (S1) and all individuals in the prediction set (S1 and S2).

3. All unknown genotypes were imputed using the procedure described above.

4. Marker effects were estimated using BayesA on the training dataset.

5. GEBVs were calculated for the prediction set, using imputed and/or low-density genotypes.

## Results

### Imputation accuracy	

The percentages of genotypes correctly imputed for each of the scenarios, considering alternative low-density genotyping strategies, are shown in Table 1. When training females were not genotyped for any SNPs, 69% of their genotypes were correctly imputed (S1). In S1, the percent of correctly imputed genotypes for prediction individuals was lower than that of the training females when the prediction individuals were not genotyped, but increased to 68% with the addition of genotyped loci. The S2 scenario had a minimum percent correctly imputed of 68% when prediction individuals were not genotyped but also increased, to 78%, with the inclusion of actual genotypes. Imputation was more successful for S2 than S1 at all genotype densities.

**Table 1:** Percent of genotypes correctly imputed in each scenario, considering alternative low-density genotyping strategies^a^.

	S1	S2
Training females – all genotypes imputed	69	

Prediction – all genotypes imputed	64	68

Prediction – all genotypes imputed, except every 10 Mb	65	73

Prediction – all genotypes imputed, except every 5 Mb	65	75

Prediction – all genotypes imputed, except every 2 Mb	68	78

^a^The number of SNPs genotyped was 55 for 10 Mb spacing, 105 for 5 Mb spacing and 251 for 2 Mb spacing.

### Genomic breeding value accuracy

**Figure 1:**
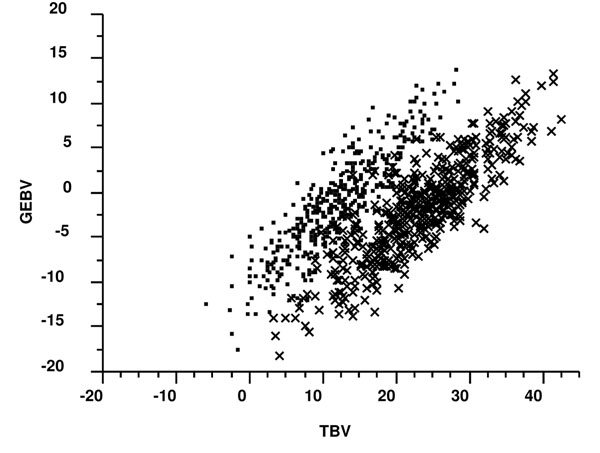
Genomic breeding values for prediction individuals when all genotypes are known (BASE scenario) versus true breeding values, where females are denoted by (•) and males by (x).

Genomic breeding value accuracy was defined as the correlation between GEBVs and true breeding values. Figure 1 depicts a plot of these values for the BASE scenario with sex of the individual identified. A sex difference in breeding values is apparent, which is assumed to result from the paternally imprinted QTL simulated in this dataset [8]. Any correlation derived from the combined data (i.e., including both sexes) would be a biased estimate of the GEBV accuracy and thus the resulting individual GEBVs were separated by sex and the correlations averaged to yield the final GEBV accuracy. The accuracies for each scenario and alternative low-density genotyping strategies are shown in Table 2. The GEBV accuracy when all genotypes were imputed was essentially the same for S1 and S2 (r=0.42) and increased in both scenarios as additional low-density genotypes were added. The maximum accuracy for S1, when SNP genotypes were included every 2 Mb, was 0.54, while the maximum accuracy for S2 was 0.66. These accuracies were compared to the BASE scenario accuracy (r=0.86), where all individuals were genotyped (Table 2). The change in accuracy ranged from -0.44 with all genotypes imputed to -0.20 for the maximum accuracy in S2 (genotypes every 2 or 5 Mb).

**Table 2:** Accuracy of genomic breeding values (r) for prediction individuals in each scenario (as the correlation between genomic breeding values and true breeding values), considering alternative low-density genotyping strategies, and change in accuracy (Δ) compared to the BASE scenario^a^.

	S1		S2	
	**r**^b^	**Δ**	**r**	**Δ**

all genotypes imputed	0.42	-0.44	0.42	-0.44

all genotypes imputed, except every 10 Mb	0.50	-0.36	0.61	-0.25

all genotypes imputed, except every 5 Mb	0.50	-0.36	0.66	-0.20

all genotypes imputed, except every 2 Mb	0.54	-0.32	0.66	-0.20

^a^BASE GEBV accuracy = 0.86

^b^The correlation was calculated separately for males and females and then averaged

## Discussion

As expected SALHI was more successful at identifying correct genotypes when additional information was added to distinguish between putative haplotypes. In prediction individuals the addition of low-density genotypes improved the ability of the imputation algorithm to discard incorrect haplotypes, while the addition of training female genotypes (in S2) improved the reliability of the geneprobs for prediction individuals and thus improved haplotype identification. The improved reliability of geneprobs was more important than the addition of low-density genotypes, as evidenced by the higher percent of correctly imputed genotypes in S2 compared to S1, but the combined information increased the percent of correctly imputed genotypes from 64% to 78% (Table 1). In contrast, the percent correctly imputed genotypes for training females (S1) did not increase with low-density genotyping in the prediction animals (results not shown). It was expected that the addition of genotypes in the females’ progeny would improve the reliability of the geneprobs, and thus improve imputation, but this information did not seem to be important for imputing genotypes in training individuals. The architecture of the population, however, would likely impact imputation success through the number of haplotypes in the library and reliability of the geneprobs. Overall computation for the creation of the haplotype library and imputation (assuming geneprobs were already calculated) was ~30 minutes per chromosome and thus this approach should scale well for larger datasets (e.g., data from the current 50-60k SNP chips in livestock).

The comparisons of GEBV accuracy using imputed genotypes with the BASE scenario provide a context for evaluating the usefulness of the current imputation approach. The GEBV accuracy when all genotypes were imputed was less than half the accuracy when all genotypes were known (Table 2) indicating that geneprobs alone were not sufficient to calculate accurate GEBVs, even when training females were genotyped. The addition of low-density genotypes, however, improved the accuracies, though the densest set (every 2 Mb) was not notably better than having SNPs every 5 Mb, in S2. The maximum accuracy using imputed genotypes was more than 20 percent smaller than the BASE GEBV accuracy, but comparisons to the accuracies of BLUP EBVs (for the prediction animals) show that improvement in breeding value accuracy can be obtained using imputed genotypes. The accuracy of BLUP EBVs for prediction individuals was 0.53 (results not shown), which is smaller than all low-density genotyping strategies in S2, indicating that even when using imputed genotypes a faster rate of genetic progress would be possible over ignoring genomic information in this dataset. The low-density panels simulated correspond to SNP densities in cattle and pigs of approximately 300, 600 or 1500 SNPs and so the cost of any decrease in GEBV accuracy from using imputed SNPs would need to be balanced against the cost of genotyping at lower densities.

Ideally a single panel of low-density SNPs is developed that has application across traits (and potentially across breeds/lines), but several issues will impact GEBV accuracy when using imputed genotypes, including the genetic architecture of the trait and the strategy for selecting SNPs to use in such panels. For traits influenced by a small number of large QTL, such as is the case in this study [8], a panel selected from SNPs associated with the trait will generally result in higher accuracy and may be preferable to a panel selected based on spacing, as done here [9]. Additionally, the GEBV accuracy using a panel based on spacing could be somewhat unpredictable across traits as some selected SNPs could be associated with a QTL by chance for some traits but have no association with QTL for others. This unpredictability can be reduced by minimizing the change in accuracy when using imputed genotypes (compared to high density) allowing for a low-density panel that would contain all of the information of a trait-specific panel of associated SNPs. For most traits in livestock production large QTL have not been identified and thus application of a single, general SNP panel is more straightforward. Results from SALHI are promising and continued improvement in the algorithm should minimize the decrease in accuracy observed here making a small SNP panel a cost-effective alternative to high-density genotyping, especially in cases where the development of trait-specific panels is not desirable.

## Conclusions

Genotype imputation using segregation analysis and a long haplotype library has promise for application in sparsely-genotyped pedigrees where low-density genotype panels applicable across traits are desired. The results of this study suggest that while genotype imputation resulted in some lost accuracy of genomic breeding values compared to a scenario where individuals were high-density genotyped, its levels of accuracy are still higher than traditional BLUP estimated breeding values. The approach presented here is under development and should scale well to current livestock genomics datasets. 	

## Competing interests

The authors declare that they have no competing interests.

## Authors' contributions

MAC performed the genotype imputation, participated in study design and drafted the manuscript. JMH performed the long range phasing and training and participated in study design. BPK helped to develop and fine-tune the imputation method. All authors read and approved the final manuscript.
